# Notes

**Published:** 1898-05-07

**Authors:** 


### NOTES.

A special Act of Parliament will be required to enable the Newcastle Corporation to provide the new site for the infirmary on the Leazes.

---

The new laboratories of the London School of Medicine for Women are to be opened in July by the Princess of Wales, who will be accompanied by the Prince of Wales.

---

It has been decided to close the Middlesex Hospital from July 17th to September 19th, for the purpose of carrying out necessary repairs and alterations and re-painting the whole building within and without. No new cases will therefore be admitted after July 1st. The cancer wards will, however, remain open, and patients will be received in them as usual. The new medical school will be opened on October 1st, and other new buildings, including the new wing for female cancer patients, will be opened early next year.

---

A curious question has arisen at Keighley as to the disposal of the body of a patient who had died in the local hospital for infectious diseases. From the remarks of the chairman at a recent meeting of the Council it appears that the body was removed from the hospital to the home of the deceased, where the corpse was lifted out of the coffin, and that, probably from that cause, there was another patient from the same house. Reference to the Infectious Diseases Prevention Act, 1890, however, will show that in all districts which have adopted that Act the sanitary authority have it in their power to prevent the removal of the body of a person who has died from infectious disease in a hospital, except for immediate interment or to a mortuary, if the medical officer of health or a registered practitioner certifies that such restriction is desirable for preventing infection.

---

Convalescent Homes are unquestionably a great boon to the sick poor. But in some instances much of the benefit is discounted by misplaced economy. Visitors at a popular South Coast seaside resort have sometimes in early spring had their attention attracted by the sight of convalescent patients bound for a well-known convalescent home, toiling up-hill for a distance of some two miles after a long journey from London, in the teeth of a bitter north-east wind, in sleet and snow, laden with parcels, and asking their way as they went, misdirected here, put on the right path there, and arriving finally at their destination utterly worn out and chilled. Surely it would be possible to arrange for a batch of patients to arrive on the same day, and for an omnibus to meet them at the station. Or, if this should prove too expensive a matter, they might surely be instructed as to the running of a local omnibus which would give them a lift on the way. A fortnight at the sea is a delightful circumstance to a sick person; but the mischief done by so long an uphill cold climb just at the first stage of recovery might well lead to very serious results, and must largely detract from the benefits received.

A grand afternoon concert, by the kind permission of the Duke and Duchess of Sutherland, will be given at Stafford House on May 11th in aid of the Special Appeal Fund for Charing Cross Hospital.

---

The Diamond Jubilee Ward—an extension of the Mansfield Hospital—has just been formally opened, the ceremony having been performed by the Duchess of Portland. A debt of nearly £400 remains on the new building.

---

The London County Council has agreed that it be referred to the Public Health Committee to inquire into the condition of the cemeteries and burial grounds within the metropolitan area, and to report whether any further provision for the purpose is necessary; and also whether it is expedient that any regulations should be laid down in respect of such places in the interest of public health and decency. There is a good deal at the back of this resolution. The whole subject sadly wants looking into—the cemeteries outside as well as those within the metropolitan area. London has spread vastly since the mass of the metropolitan graveyards were closed in 1852, and many of the then suburban cemeteries are now surrounded by a considerable population.

---

An inquest held last Saturday on a young man who had died from phosphorus necrosis should draw attention afresh to the duty of notifying certain cases of disease to the Chief Inspector of Factories, which is now laid upon all medical practitioners—a duty of which, we fancy, many are quite ignorant. According to the report of the inquest given in the *Daily Chronicle*, the Coroner said, "I should never have heard of the case if it had not been for the Registrar." From this it would appear that either no report of the case had been sent to the factory inspector or the officials at the Home Office had failed to follow up the case. We would then point out that, besides notifying under what is known as the Notification Act, which has to do with the sanitary authorities, medical men are bound to notify certain cases of disease in quite another quarter. In the Factory and Workshops Act, 1895, it is laid down—Section 27 (1)—that "every medical practitioner attending on or called in to visit a patient whom he believes to be suffering from lead, phosphorus, or arsenical poisoning, or anthrax, contracted in any factory or workshop shall . . . send to the Chief Inspector of Factories at the Home Office, London, a notice stating the name and full postal address of the patient, and the disease from which, in the opinion of the medical practitioner, the patient is suffering," the medical man being entitled to a fee of 2s. 6d. for each such notification. It is, perhaps, a pity that this clause ever got into the Act, as all such sanitary notifications should go primarily to the sanitary authority; but there it is, and it must be obeyed. Possibly the growing sanitary responsibility of the Factory Department of the Home Office was one reason why Dr. Whitelegge was appointed Chief Inspector of Factories. In any case, the fact that there is a medical head to this department takes away many of the objections which some might feel to notifying to a lay official.

